# A Multiparametric Protocol for the Detailed Phytochemical and Antioxidant Characterisation of Plant Extracts

**DOI:** 10.3390/mps6020040

**Published:** 2023-04-05

**Authors:** Anna Michalaki, Konstantinos Grintzalis

**Affiliations:** School of Biotechnology, Dublin City University, D09 Y5NO Dublin, Ireland

**Keywords:** phytochemicals, biochemical assays, herbal plants, medicinal plants, polyphenols, tannins, flavonoids, antioxidant scavenging, dpph, abts, galvinoxyl radical, metal reduction

## Abstract

Medicinal and herbal plants are abundant sources of phytochemicals, which are biologically active compounds with potential health benefits. The characterisation of phytochemicals has been the subject of many studies, but there is a lack of comprehensive assays to accurately assess the main phytochemical categories and their antioxidant properties. To address this, the present study has developed a multiparametric protocol comprising eight biochemical assays, which quantify the major categories of phytochemicals, including polyphenols, tannins and flavonoids, as well as their antioxidant and scavenging potential. The presented protocol offers several advantages over other methods, including higher sensitivity and significantly lower cost, making it a simpler and more affordable approach compared to commercial kits. The protocol was tested on two datasets with seventeen distinct herbal and medicinal plants, and the results demonstrated its effectiveness in accurately characterising the phytochemical composition of plant samples. The modular design of the protocol allows its adaptation to any spectrophotometric instrumentation, while all assays are simple to follow and require a minimum number of analytical steps.

## 1. Introduction

Since ancient times, plants provide mankind with many remedies and food supplements, and in some countries, medicinal plants may be the primary or sole source of healthcare [1,2]. Some examples of medicinal and herbal plant products include chamomile, ephedra, garlic, ginseng, marijuana and opium. Furthermore, plants have a significant impact on diet in both societal (i.e., veganism) but also in practical contexts in relation to human physiology. The nutritional and medicinal benefits of plants are reflected in their phytochemical components, as plants are able to synthesise a large range of compounds known as phytochemicals [3,4,5]. The metabolites of plants are categorised into two groups: primary and secondary metabolites. The primary metabolites are involved in the major metabolic pathways for plant growth and development, while secondary metabolites serve non-essential purposes in the plants [6,7]. Secondary metabolites are required for plants to survive as these compounds are involved in adaptation and survival mechanisms, but also have been proven to have medicinal properties [4,8]. As a result, medicinal and herbal plants play a critical role in the food and pharmaceutical industries [9]. 

Several in vivo and in vitro studies have been focused on the beneficial biological actions of plant extracts highlighting their importance in ethnobotany. Most innovative studies emphasise the phytochemical characterisation and identification of biologically active compounds, which can then be synthesised in organic chemistry laboratories or in heterologous hosts [10]. In this effort, several assays for the determination of the major categories of phytochemicals (i.e., polyphenols, tannins, flavonoids, alkaloids, etc.) were combined with assays focusing on the assessment of the antioxidant properties of plant extracts. These methods are useful for the identification of bioactive compounds. To date, there is not an optimised comprehensive protocol to provide a series of analytical options within a general (not detailed) phytochemical characterisation. 

**Figure 1:**
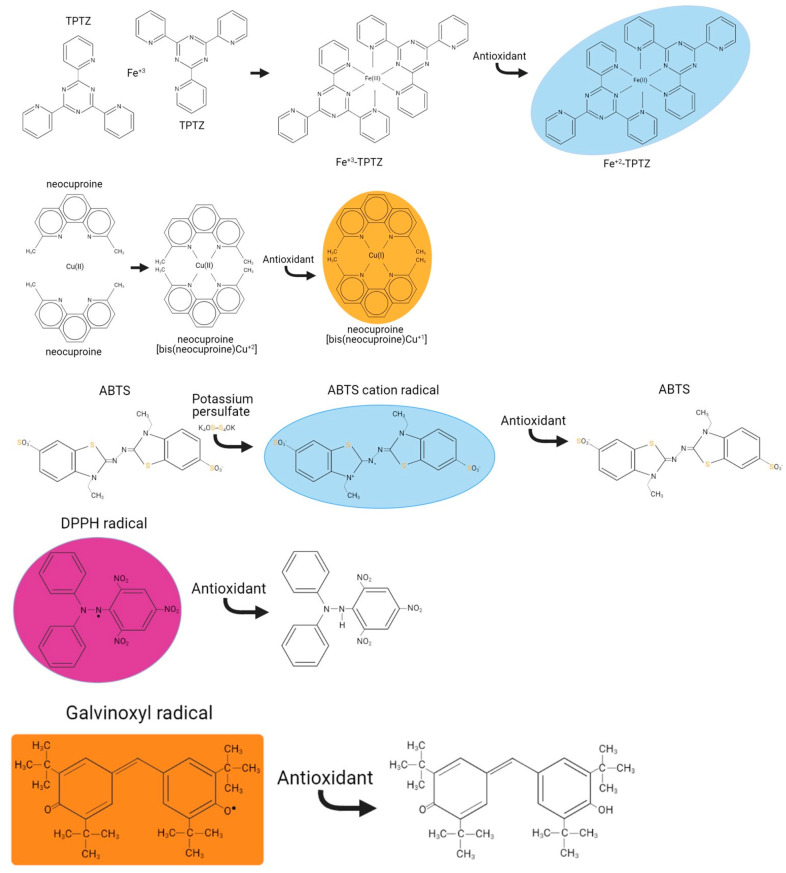
The metal reduction and radical scavenging assays.

The present multiparametric protocol provides targeted assays for the characterisation of the main categories of plant metabolites for eight distinct biochemical protocols with standardised microplate assays. Specifically, flavonoids are quantified by their reaction with aluminium trichloride [11], tannins are assayed by their reaction with vanillin under acidic conditions [12], and polyphenols are assessed by the Folin reagent [13]. In relation to the metal scavenging [14] and the antioxidant potential [15] of plant extracts, the capacity of plant extracts to reduce ions such as iron and copper (Figure 1) was based on assays using the 2,4,6-tri-pyridyl-s-triazine [16] and neocuproine reagents [17], respectively. Both assays require first the metal cation to generate a complex with the reagent, followed by the reduction of the metal cation to generate a chromogenic reduced metal-reagent complex. Finally, assays based on the reduction of the stable DPPH [18], ABTS^•+^ cation [19] and galvinoxyl radicals are performed using the loss of their colour when scavenged by a phytochemical extract, to assess the % of scavenging capacity (Figure 1). Overall, the protocols presented underwent extensive troubleshooting and optimisation of the conditions existing in the original methods, previously applied on extracts from the sumac plant [20] aiming to provide a unified gold standard approach. 

## 2. Experimental Design

**Figure 2:**
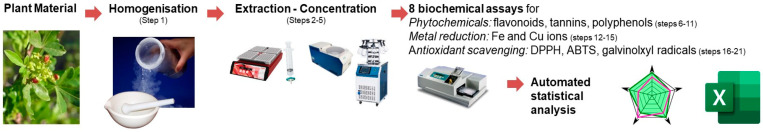
General outline of the steps of the multiparametric protocol.

The protocol procedures (outlined in Figure 2), begin with the initial processing of the plant material (step 1) and the extraction of plant metabolites followed by their subsequent concentration (steps 2–5). All assays (steps 6–24) can be performed in any sequence and are independent of each other. 

### 2.1. Materials

1. Aluminium chloride anhydrous (AlCl_3_) (Alfa Aesar, cat. no. L18489.22)
2. Ammonium acetate (Fisher Scientific, cat. no. A/3440/60)
3. 2,2′-azino-bis-3-ethylbenzothiazoline-6-sulphonic acid (ABTS^•+^) (Sigma, cat. no. A1888)
4. Catechin hydrate (Sigma, cat. no. 22110)
5. Chloroform, HPLC grade (ACROS Organics, cat. no. 158210010) CAUTION! Chloroform fumes are hazardous to the skin, eyes and airways. It should be handled in a fume hood with appropriate protective equipment, including eye protection and gloves.
6. Copper sulphate pentahydrate (CuSO_4_.5H_2_O) (Sigma, cat. no. 209198)
7. 2,2-diphenyl-1-picrylhydrazyl (DPPH) (Sigma, cat. no. 14357)
8. Ferric chloride hexahydrate (FeCl_3_.6H_2_O) (Sigma, cat. no. F2877)
9. Folin–Ciocalteu’s reagent (VWR, cat. no. 31360.264)
10. Gallic acid (Sigma, cat. no. G7384)
11. Glacial acetic acid (Sigma, cat. no. 338826) CAUTION! Acetic acid fumes are hazardous to the skin, eyes and airways. It should be handled in a fume hood with appropriate protective equipment, including eye protection and gloves. Acetic acid is highly corrosive.
12. Hydrochloric acid (HCl) (Fisher Scientific, cat. no. H/1150/PB17) CAUTION! Hydrochloric acid fumes are hazardous to the skin, eyes and airways. It should be handled in a fume hood with appropriate protective equipment, including eye protection and gloves. Hydrochloric acid is highly corrosive.
13. Methanol, HPLC-MS grade (Honeywell, cat. no. 34966) CAUTION! Methanol is highly flammable and hazardous to the skin, eyes and airways.
14. Neocuproine hydrochloride (ACROS Organics, cat. no. 153310050)
15. Potassium persulphate (Sigma, cat. no. 379824)
16. Sodium acetate anhydrous (Sigma, cat. no. S8750)
17. Sodium carbonate (Na_2_CO_3_) (Sigma, cat. no. S7795)
18. Sodium hydroxide (NaOH) (Sigma, cat. no. S5881)
19. Sodium nitrite (NaNO_2_) (Sigma, cat. no. V8012-50UG)
20. Sulphuric acid (H_2_SO_4_) (ACROS Organics, cat. no. 424525000) CAUTION! Sulfuric acid is hazardous to the skin, eyes and airways. It should be handled in a fume hood with appropriate protective equipment, including eye protection and gloves. Sulphuric acid is highly corrosive.
21. 2,4,6-tri-pyridyl-s-triazine (TPTZ) (Sigma, cat. no. 93285)
22. Vanillin (Sigma, cat. no. V1104)
23. Water H_2_O, HPLC-MS grade (Merck, cat. no. 1.15333.1000)

### 2.2. Equipment

- Syringe filters (0.2 μm; Thermo Fisher Scientific, Ireland cat. no. 723–2520)
- Microplate reader (TECAN)
- Centrifuge for Eppendorf tubes

## 3. Procedure 

### 3.1. Plant Homogenisation, Extraction and Preparation for Assays (1 h)

1. Collect plants (i.e., leaves, roots, stems, flowers) and either quench by immediate freezing in liquid nitrogen or homogenise tissue, i.e., cutting tissues in slices and oven-drying at 40 °C followed by grinding [20,21].
2. Extract plant tissues in HPLC-MS methanol:HPLC-MS water (80%:20% *v*/*v*) with vigorous vortexing to facilitate the extraction of polar molecules. This approach is sufficient to extract different categories of organic and polar molecules, however, it can be tailored to the applications of the laboratory.
3. Clear the extract from debris by centrifugation at 18,000× *g* at 4 °C for 10 min and collect the clear supernatant. An alternative approach for removing debris is filtration using a syringe filter (0.45 μm filter membrane).
4. The plant extract can be used immediately or concentrated by speed vacuum evaporation/lyophilisation. The extract can be aliquoted prior to use or drying at this point. For reference to the initial amount of plant tissue extracted, a record of the plant material collected, and the volume of extraction solvent used must be kept.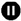 **PAUSE STEP** The extracted samples after evaporation are stable at −80 °C. Prior to analysis, the evaporated samples should be reconstituted.
6. Prepare serial dilutions of the methanolic extracts in ddH_2_O and use for all assays.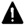 **CRITICAL STEP** Only for the assessment of galvinoxyl radical scavenging the dilutions prepared in methanol citrate.
8. If the extract (from step 4) was evaporated/lyophilised, then it is first reconstituted in ddH_2_O or other solvents with vigorous vortexing and filtering to remove undissolved particulates, and then appropriately diluted in ddH_2_O to avoid any interferences.

### 3.2. Quantification of Flavonoids (0.5 h)

- Samples (and catechin standards 10–100 μΜ) are assayed for flavonoids according to Table 1.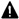 **CRITICAL STEP** Agitate by pipetting to solubilise mixture aggregates.
- Incubate mixtures for 10 min at room temperature and measure absorbance at 500 nm. The net absorbance derived from the absorbance difference of sample/standard minus reagent blank is converted to equivalents of catechin nmoles using the corresponding standard curve.

### 3.3. Quantification of Tannins (0.5 h)

- Samples (and catechin standards 10–100 μΜ) are assayed for tannins according to Table 2.

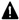
 **CRITICAL STEP** Agitate by pipetting to solubilise mixture aggregates.
- Incubate mixtures for 10 min at room temperature and measure absorbance at 500 nm. The net absorbance derived from the absorbance difference of sample/standard minus reagent blank is converted to equivalents of catechin nmoles using the corresponding standard curve.

### 3.4. Quantification of Polyphenols (1 h)

- Samples (and gallic acid standards 10–100 μΜ) are assayed for polyphenols according to Table 3.

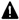
 **CRITICAL STEP** Agitate by pipetting to ensure homogeneity.
- Incubate mixtures for 40 min at room temperature and measure absorbance at 765 nm. The net absorbance derived from the absorbance difference of sample/standard minus reagent blank is converted to equivalents of gallic acid nmoles using the corresponding standard curve.

### 3.5. Quantification of Ferric Reducing Power (FeRP) (1 h)

- Samples (and gallic acid standards 10–100 μΜ) are assayed for ferric-reducing power according to Table 4.

- Incubate mixtures for 40 min at room temperature and measure absorbance at 595 nm. The net absorbance derived from the absorbance difference of sample/standard minus reagent blank is converted to equivalents of gallic acid nmoles using the corresponding standard curve.

### 3.6. Quantification of Cupric Reducing Power (CuRP) (1 h)

- Samples (and gallic acid standards 10–100 μΜ) are assayed for cupric-reducing power according to Table 5.

- Incubate mixtures for 40 min at room temperature and measure absorbance at 450 nm. The net absorbance derived from the absorbance difference of sample/standard minus reagent blank is converted to equivalents of gallic acid nmoles using the corresponding standard curve.

### 3.7. Quantification of DPPH Radical Scavenging (1 h)

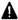
 **CRITICAL STEP** Before starting the experiment, a fresh DPPH reagent at ~1.3 A 515 nm is prepared in methanol:acetic acid pH 5.5 (8:2). Ensure that mixing 200 μL of this DPPH stock is with 100 μL ddH_2_O as reagent blank gives an absorbance of ~0.8 at 515 nm [22]. The reason being is that the absorbance of the reagent blank (RB) will be the initial 100% radical which needs to be in the linear absorbance scale of the microplate reader used and also in excess to allow sufficient scavenging. If this is not the case adjust the dilution/concentration of the DPPH stock.
- Samples (and catechin standards 5–100 μΜ) are assayed for DPPH radical scavenging capacity according to Table 6.

- Incubate mixtures for 10 min at room temperature protected from light and measure absorbance at 515 nm. Calculate the % of DPPH radical scavenging as follows:
- 100 × [(A_ReagentBlank_−A_SolventBlank_)−(A_Sample/Standard_−A_SolventBlank_)]/(A_ReagentBlank_−A_SolventBlank_). The % of DPPH radical scavenging is converted to equivalents of catechin nmoles using the corresponding standard curve.

### 3.8. Quantification of ABTS Radical Cation (ABTS^•+^) Scavenging (1 h)

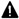
 **CRITICAL STEP** Before starting the experiment, a fresh ABTS^•+^ reagent at ~1.3 A 734 nm is prepared in ddH_2_O. Ensure that mixing 200 μL of this ABTS^•+^ stock with 100 μL ddH_2_O (will be used as reagent blank) gives an absorbance of ~0.8 at 734 nm. The reason being is that the absorbance of the reagent blank will be the initial 100% radical which needs to be in the linear absorbance scale of the microplate reader used and also in excess to allow sufficient scavenging. If this is not the case, adjust the dilution/concentration of the ABTS^•+^ stock.
- Samples (and catechin standards 5–100 μΜ) are assayed for ABTS^•+^ radical scavenging capacity according to Table 7.

- Incubate mixtures for 40 min at room temperature protected from light and measure absorbance at 734 nm. Calculate the % of ABTS^•+^ radical scavenging as follows:
- 100 × [(A_ReagentBlank_−A_SolventBlank_)−(A_Sample/Standard_−A_SolventBlank_)]/(A_ReagentBlank_−A_SolventBlank_). The % of ABTS radical scavenging is converted to equivalents of catechin nmoles using the corresponding standard curve.

### 3.9. Quantification of Galvinoxyl Radical Scavenging (1 h)

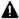
 **CRITICAL STEP** Before starting the experiment, a fresh galvinoxyl radical reagent at ~1.3 A 435 nm is prepared in methanol:citric acid pH 6 (9:1). Ensure that mixing 200 μL of this galvinoxyl radical stock is with 100 μL methanol:citric acid pH 6 (9:1) as reagent blank gives an absorbance of ~0.8 at 435 nm. The reason being is that the absorbance of the reagent blank (RB) will be the initial 100% radical which needs to be in the linear absorbance scale of the microplate reader used and also in excess to allow sufficient scavenging. If this is not the case adjust the dilution/concentration of the galvinoxyl radical stock.
- Samples (and catechin standards 5–100 μΜ) are assayed for galvinoxyl radical scavenging capacity according to Table 8.

- Incubate mixtures for 10 min at room temperature protected from light and measure absorbance at 435 nm. Calculate the % of galvinoxyl radical scavenging as follows:
- 100 × [(A_ReagentBlank_−A_SolventBlank_)−(A_Sample/Standard_−A_SolventBlank_)]/(A_ReagentBlank_−A_SolventBlank_). The % of galvinoxyl radical scavenging is converted to equivalents of catechin nmoles using the corresponding standard curve.

### 3.10. Troubleshooting

- All protocols presented are straightforward with minimum number of steps similar to kit-based assays. A troubleshooting table (Table 9) is presented for possible caveats.

## 4. Expected Results

**Figure 3:**
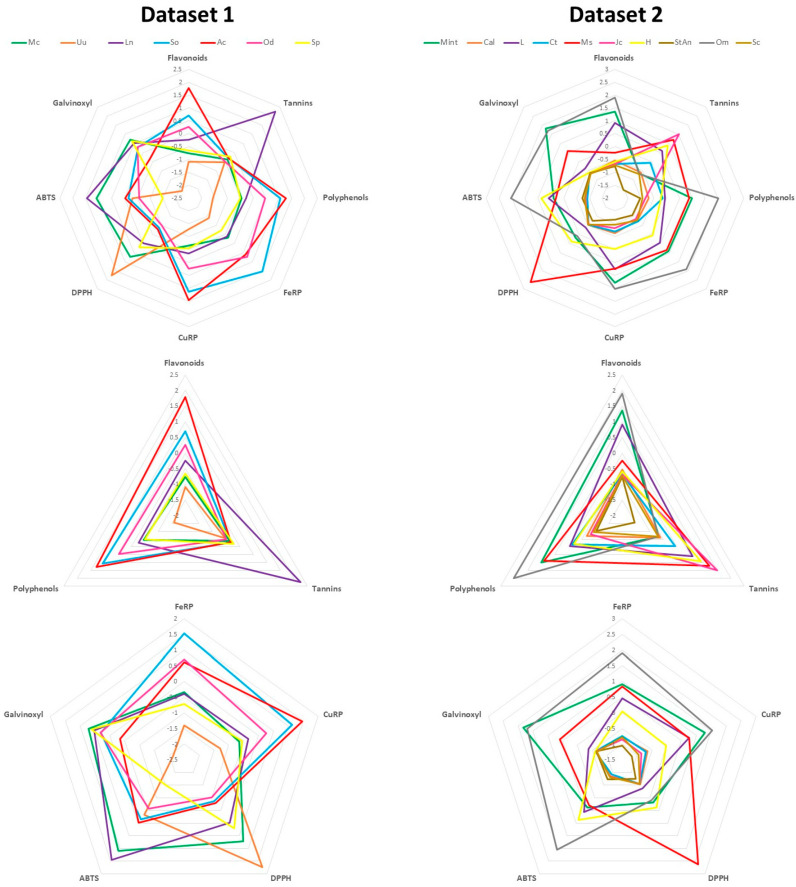
Radar charts of results. Abbreviations: *Matricaria chamomilla* (Greek chamomile, Mc), *Urtica urens* (stinging nettle, Uu), *Laurus nobilis* (laurel or sweet bay, Ln), *Salvia officinalis* (sage, So), *Aloysia citrodora* (lemon verbena, Ac), *Origanum dictamnus* (dittany, Od), *Sideritis perfoliata* (Greek mountain tea, Sp). Mint, calendula (Cal), lavender (L), *Clitoria ternatea* (Ct), *Myosotis sylvatica* (forget me not flower, Ms), *Juniperus communis* (juniper berry, Jc), *Hibiscus* (H), star anise (StAn), *Origanum majorana* (Om), *Schisandra chinensis* (Sc).

The multiparametric protocol described here can be applied to any type of plant material. Previously, we demonstrated this approach in our article on the sumac root, leaf and stem extracts on an in vitro ethanol toxicity model system [20]. In this study, we provide the biochemical analysis of Greek herbal plant extracts (dataset 1) and other common plants (dataset 2) summarised as indicative exemplars of our outlined approach in Supplementary Excel files with automated calculations to assist the end user. Each sheet contains a respective protocol in addition to a standard curve and sample analysis (for four replicates per samples). The technical reproducibility of the methods presented is reflected on the low coefficient of variance obtained from independent replicates of each sample. This is a result of the simplicity of each assay performed in a minimum number of steps. Simple statistics with unpaired tests can be performed using the Excel files provided. Furthermore, the results can be collectively processed (in their averages per sample) after z-score standardisation to avoid the large differences among data skewing the findings. The results are easily presented in radar charts (Figure 3), which can collectively summarise the parameters measured for a great number of plants. Values were z-score standardised as outlined in the automated Excel file. In the first dataset of the Greek herbal plants, the stinging nettle (*Urtica urens*, Uu) is the sample in the inner center of the radar charts, thus with the minimum amount of all measurement parameters, while for other samples characteristic increased values such as tannins for *Laurus nobilis* (Ln) can be easily visualised. 

Although there are many biochemical methods available for determining the antioxidant activities of biological materials [27], to our knowledge there has been no attempt so far in the research community to generate a gold standard approach on the outlined topic of a general characterisation of plant extracts. Furthermore, as a general rule for the assays available in the literature, these mostly rely on large reagent and sample volumes, which in turn results in increased consumption of reagents, generating additional lab waste and requiring more samples, which, in turn, would reflect on the sensitivity of the methods [28]. The methods developed here aim to quickly, accurately, and with the highest sensitivity provide results for the general characterisation of plants. 

The protocols presented here provide a detailed characterisation of plant extract as their primary focus to identify the main phytochemical categories. However, the specific identification of compounds would require significant and cumbersome analytical instrumentation such as chromatographic separation coupled with mass spectrometry or NMR spectroscopy to achieve the specific elucidation of plant extract chemical composition [29]. Although there have been many advances in hyphenated analytical methods for the quantification and identification of polyphenols, flavonoids and tannins, providing their analytical coverage is limited by matrix effects and the cost of analytical equipment [30]. The protocol presented here could also be part of a more detailed assessment if a user chooses to reconstitute the samples from step 4 following their extraction and proceed towards an HPLC-MS analysis. 

All methods presented here have been modified extensively from their original studies and were further modulated and optimised to promote an analytical approach with the least number of steps and for processing large sample numbers, with results comparable in reproducibility, accuracy and applicability to a number of kits reviewed. The protocols presented result in significant time saving, reducing the total cost for reagents and providing a cost-effective method. As an example, the typical kits available would be limited to usually 200 samples and cost approximately 500 euros for FeRP and polyphenols. The flexibility of the multiparametric protocol also highlights its versatility as different parameters can be handled and the workload can be spread appropriately, or users can focus only on specific methods of their research interest. The feasibility of this protocol is reflected in the Supplementary Excel files, which provide worked-out examples for the fast processing of results generated. All calculations and analyses are easily performed in an automated fashion which allows the user to generate their quantitative results. We foresee the application of this method to the wider research community and a diverse set of research themes such as phytochemistry, toxicology and redox biochemistry. 

## 5. Conclusions

In summary, the present multiparametric protocol provides a comprehensive and novel approach to assessing the phytochemical and antioxidant properties of plant and food extracts. The methods described here have been explored in the literature extensively with different elaborations and approaches followed as independent assays and yet never as a unified multiparametric approach. The protocol employs eight parameters that cover the three major plant metabolite categories as well as the antioxidant status of plant extracts. While maintaining a high level of accuracy and sensitivity, this approach has the potential to significantly reduce the cost and time related to traditional methods. Furthermore, the protocol is highly adaptable as to allow researchers to focus on specific parameters of interest. The automated calculations and standardised reporting presented in this study improve the reproducibility and reliability of the approach. To our knowledge, this is the first approach of multiple assay protocols which can provide a holistic description of eight parameters covering the three main phytochemical categories of plant metabolites and the antioxidant status of plant extracts. 

## 6. Reagents Setup

Reagents are listed in order of appearance for each protocol.

**Amount of 2% NaNO_2_:** Prepare fresh by dissolving 200 mg NaNO_2_ in 10 mL ddH_2_O.

**Amount of 7.5% AlCl_3_:** Prepare fresh by dissolving 750 mg AlCl_3_ in 10 mL ddH_2_O. **!CAUTION** Dissolution is exothermal and produces gas and all tasks must be performed in the fume hood. 

**Amount of 3.5 M NaOH:** Dissolve 14 g NaOH (MW: 40 g/mol) in 100 mL ddH_2_O. 

**Amount of 4% vanillin:** Prepare fresh by dissolving 400 mg vanillin in 10 mL methanol. 

**Amount of 100% H_2_SO_4_:** CAUTION! H_2_SO_4_ is concentrated acid and all handling is performed in the fume hood.

**Amount of 4x Folin reagent:** Prepare fresh by diluting the Folin reagent 4x with ddH_2_O. 

**Amount of 1.89 M Na_2_CO_3_:** Dissolve 10 g Na_2_CO_3_ (MW: 105.99 g/mol) in 50 mL ddH_2_O. 

**Amount of 300 mM acetic acid:** Prepare fresh by dissolving 0.093 g sodium acetate anhydrous (MW: 82.03 g/mol) and 0.8 mL glacial acetic acid in 49.2 mL ddH_2_O. 

**Amount of 40 mM HCl:** Diluting the concentrated 37% (or 12 M) HCl 300x with ddH_2_O by mixing 150 mL ddH_2_O with 0.5 mL 12 M HCl under stirring. CAUTION! HCl is a concentrated fumigous acid and all handling must be performed in the fume hood. 

**TPTZ:** Prepare fresh by dissolving 3.12 mg TPTZ (MW: 312.33 g/mol) in 500 μL methanol. Then add 500 μL 40 mM HCl. 

**Amount of 0.54% FeCl_3_.6H_2_O:** Prepare fresh by dissolving 54 mg ferric chloride hexahydrate in 10 mL ddH_2_O. 

**FeRP reagent:** Mix TPTZ:0.54% FeCl_3_.6H_2_O:300 mM acetic acid in a ratio of 1:1:10. This complex form of iron when reduced will absorb at 595 nm (Figure 1). 

**Amount of 10 mM Cu^+2^:** Prepare fresh by dissolving 24.9 mg copper sulphate pentahydrate (MW: 249.68 g/mL) in 10 mL ddH_2_O. 

**Amount of 6 mM neocuproine:** Prepare fresh by dissolving 16 mg neocuproine (MW: 262.73 g/mL) in 10 mL ddH_2_O. 

**1M ammonium acetate:** Prepare fresh by dissolving 3.86 g ammonium acetate (MW: 77.08 g/mol) in 25 mL ddH_2_O and after complete dissolution, adjust volume to 50 mL. **!CAUTION** The solid ammonium acetate will occupy some volume of the solution. 

**Cu-neocuproine-ammonium acetate reagent:** Mix 10 mM Cu^+2^, 6 mM neocuproine and 1 M ammonium acetate in equal volumes (1:1:1). This complex form of copper when reduced will absorb at 450 nm (Figure 1). 

**Amount of 100 mM acetic acid pH 5.5:** Prepare fresh by dissolving 0.42 g sodium acetate anhydrous (MW: 82.04 g/mol) in ddH_2_O. Adjust the pH to 5.5 and the final volume to 50 mL. 

**Methanol:100 mM acetic acid pH 5.5 (9:1):** Prepare fresh by mixing 80 mL methanol with 20 mL 100 mM acetic acid pH 5.5. 

**DPPH radical:** Prepare fresh by dissolving 25 mg DPPH (MW: 394.32 g/mol) in 10 mL methanol. Dilute this stock with methanol:acetic acid pH 5.5 (8:2) and filter (0.22 μM). Dilute further this stock with methanol:acetic acid pH 5.5 (8:2) to a value of ~1.3A at 515 nm. This value is set to be in the linear range of the spectrophotometer/microplate when diluted 1.5× in the assay (mixing 200 μL from the DPPH with 100 μL ddH_2_O). DPPH is a stable radical which absorbs at 515 nm and when scavenged decolourises (Figure 1). 

**Amount of 14 mM ABTS radical cation (ABTS^•+^):** Prepare fresh by dissolving 77 mg ABTS (MW: 548.68 g/mol) in 10 mL ddH_2_O. 

**Amount of 5 mM potassium persulphate:** Prepare fresh by dissolving 13.5 mg potassium persulphate (MW: 270.32 g/mol) in 10 mL ddH_2_O. 

**ABTS radical cation (ABTS^•+^):** Mix the 14 mM ABTS and the 5 mM potassium persulphate reagents 1:1 and incubate in the dark at RT for 12 h before use. ABTS^•+^ is a stable radical formed by potassium persulphate radical initiation. Dilute further this stock with ddH_2_O to a value of ~1.3 A at 734 nm. This value is set to be in the linear range of the spectrophotometer/microplate when diluted 1.5× in the assay (mixing 200 μL from the ABTS radical cation with 100 μL ddH_2_O). ABTS^•+^ is a stable radical formed by potassium persulphate radical initiation which absorbs at 734 nm and when scavenged decolourises (Figure 1). 

**Amount of 100 mM citric acid pH 6:** Prepare fresh by dissolving 0.1921 g sodium acetate anhydrous (MW: 192.12 g/mol) in ddH_2_O. Adjust the pH to 6 and the final volume to 100 mL. 

**Methanol:100 mM citric acid pH 6 (9:1):** Prepare fresh by mixing 90 mL methanol with 10 mL 100 mM citric acid pH 6. 

**Galvinoxyl radical:** Prepare fresh by dissolving 50 mg DPPH (MW: 394.32 g/mol) in 10 mL methanol. Dilute this stock with methanol:citric acid pH 6 (9:1) and filter (0.22 μM). Dilute further this stock with methanol:citric acid pH 6 (9:1) to a value of ~1.3 A at 435 nm. This value is set to be in the linear range of the spectrophotometer/microplate when diluted 1.5x in the assay [mixing 200 μL from the galvinoxyl radical with 100 μL methanol:citric acid pH 6 (9:1)]. Galvinoxyl radical is a stable radical which absorbs at 435 nm and when scavenged decolourises (Figure 1). 

### Standard Curves for Assays 

**Catechin:** Prepare a 30 mM stock solution in methanol by dissolving 8.79 mg catechin (MW: 290.27 g/mol) in 1 mL methanol. Dilute the 30 mM stock 300x to 100 μM and follow to 10 μM in ddH_2_O. Prepare a series of dilutions of standards from each stock for 1–10 μM and 10–100 μΜ, respectively, for the linear standard curves. 

**Gallic acid:** Prepare a 10 mM stock solution by dissolving 17.1 mg gallic acid (MW: 170.12 g/mol) in 10 mL methanol. Dilute the 10 mM stock solution to 100 μM and follow to 10 μM in ddH_2_O. Prepare a series of dilutions of standards from each stock for 1–10 μM and 10–100 μΜ, respectively, for the linear standard curves. 

## Figures and Tables

**Table 1:** Flavonoids assay conditions.

Reagents	Reagent Blank (μL)	Sample/Standard (μL)
ddH_2_O	100	-
Sample diluted in ddH_2_O or catechin standard	-	100
2% NaNO_2_	50	50
Incubate 10 min at RT		
7.5% AlCl_3_	50	50
3.5 N NaOH	50	50

**Table 2:** Tannins assay conditions.

Reagents	Reagent Blank (μL)	Sample/Standard (μL)
ddH_2_O	100	-
Sample diluted in ddH_2_O or catechin standard	-	100
4% vanillin in methanol	100	100
18 M H_2_SO_4_	50	50

**Table 3:** Polyphenols assay conditions.

Reagents	Reagent Blank (μL)	Sample/Standard (μL)
ddH_2_O	100	-
Sample diluted in ddH_2_O or gallic acid standard	-	100
4× Folin reagent	100	100
1.89 M Na_2_CO_3_	100	100

**Table 4:** Ferric-reducing power (FeRP) assay conditions.

Reagents	Reagent Blank (μL)	Sample/Standard (μL)
ddH_2_O	125	-
Sample diluted in ddH_2_O or gallic acid standard	-	125
FERP reagent	125	125

**Table 5:** Cupric-reducing power (CuRP) assay conditions.

Reagents	Reagent Blank (μL)	Sample/Standard (μL)
ddH_2_O	125	-
Sample diluted in ddH_2_O or gallic acid standard	-	125
Cu-neocuproine–ammonium acetate reagent	125	125

**Table 6:** DPPH radical scavenging assay conditions.

Reagents	Solvent Blank (μL)	Reagent Blank (μL)	Sample/Standard (μL)
ddH_2_O	100	100	-
Methanol:acetic acid pH 5.5 (8:2)	200	-	-
Sample diluted in ddH_2_O or catechin standard	-	-	100
DPPH radical appropriately dilutedin methanol:acetic acid pH 5.5 (8:2)	-	200	200

**Table 7:** ABTS radical cation scavenging assay conditions.

Reagents	Solvent Blank (μL)	Reagent Blank (μL)	Sample/Standard (μL)
ddH_2_O	300	100	-
Sample diluted in ddH_2_O or catechin standard	-	-	100
ABTS^•+^ radical appropriately diluted in ddH_2_O	-	200	200

**Table 8:** Galvinoxyl radical cation scavenging assay conditions.

Reagents	Solvent Blank (μL)	Reagent Blank (μL)	Sample/Standard (μL)
Methanol:0.1 M citrate pH 6 (90:10)	300	100	-
Sample appropriately diluted in methanol:0.1 M citrate pH 6 (90:10) or catechin standard	-	-	100
Galvinoxyl radical cation appropriately diluted in methanol:0.1 M citrate pH 6 (90:10)	-	200	200

**Table 9:** Troubleshooting and caveats.

Problem	Possible Reason	Solution [23,24,25,26]
No signal detected in sample	Too low levels of parameter quantified or very high dilution	Extract more plant sample per mL solvent or concentrate more sample material by evaporation
Too high signal in the non-linear range	Too high levels of parameter quantified in sample	Dilute the samples appropriately with ddH_2_O to be in the linear range of the method
Not available plate but only cuvette spectrophometer is available	Adaptability of the methods to different volume	All protocols are designed for microplate setting as this approach is miniaturised and faster. In the event that a plate reader is not available, all reactions can be scaled up for cuvette using a spectrophotometer
High variability among biological replicates	Actual biological variability in the individual plant samples	Increase the number of biological replicates

## Data Availability

All data for this study are provided in the Supplementary as worked-out examples of the protocol.

## Supplementary Materials

The following supporting information can be downloaded at: https://www.mdpi.com/article/10.3390/mps6020040/s1, Excel File Dataset 1, Excel File Dataset 2. The supplementary datasets provide worked out examples with all calculations automated for all protocols presented with their standard curves and the final radar charts. These files will assist the reader to perform fast their calculations. Dataset 1 covers the Greek herbal plant extracts and dataset 2 other common plants.
